# In vitro hyperspectral biomarkers of human chondrosarcoma cells in nanoparticle-mediated radiosensitization using carbon ions

**DOI:** 10.1038/s41598-023-41991-9

**Published:** 2023-09-09

**Authors:** Mihaela Tudor, Roxana Cristina Popescu, Raluca D. Negoita, Antoine Gilbert, Mihaela A. Ilisanu, Mihaela Temelie, Anca Dinischiotu, François Chevalier, Mona Mihailescu, Diana Iulia Savu

**Affiliations:** 1 Department of Life and Environmental Physics, Horia Hulubei National Institute for R&D in Physics and Nuclear Engineering, Reactorului 30, P.O. Box MG-6, 077125 Magurele, Romania; 2 Faculty of Biology, University of Bucharest, Splaiul Independentei 91-95, 050095 Bucharest, Romania; 3 Department of Science and Engineering of Oxide Materials and Nanomaterials, Politehnica University of Bucharest, Gheorghe Polizu Street, 1-7, 011061 Bucharest, Romania; 4 Applied Sciences Doctoral School, Politehnica University Bucharest, Bucharest, Romania; 5 UMR6252 CIMAP, Team Applications in Radiobiology with Accelerated Ions, CEA—CNRS—ENSICAEN—Université de Caen Normandie, 14000 Caen, France; 6 Doctoral School of Computer Sciences, Politehnica University Bucharest, Bucharest, Romania; 7 Holographic Imaging and Processing Laboratory, Physics Department, Politehnica University Bucharest, Bucharest, Romania; 8 Centre for Research in Fundamental Sciences Applied in Engineering, Politehnica University Bucharest, Bucharest, Romania

**Keywords:** Biological techniques, Cancer, Cell biology, Biomarkers, Medical research, Oncology, Engineering, Nanoscience and technology

## Abstract

New therapeutic approaches are needed for the management of the highly chemo- and radioresistant chondrosarcoma (CHS). In this work, we used polyethylene glycol-encapsulated iron oxide nanoparticles for the intracellular delivery of the chemotherapeutic doxorubicin (IONP_DOX_) to augment the cytotoxic effects of carbon ions in comparison to photon radiation therapy. The in vitro biological effects were investigated in SW1353 chondrosarcoma cells focusing on the following parameters: cell survival using clonogenic test, detection of micronuclei (MN) by cytokinesis blocked micronucleus assay and morphology together with spectral fingerprints of nuclei using enhanced dark-field microscopy (EDFM) assembled with a hyperspectral imaging (HI) module. The combination of IONP_DOX_ with ion carbon or photon irradiation increased the lethal effects of irradiation alone in correlation with the induction of MN. Alterations in the hyperspectral images and spectral profiles of nuclei reflected the CHS cell biological modifications following the treatments, highlighting possible new spectroscopic markers of cancer therapy effects. These outcomes showed that the proposed combined treatment is promising in improving CHS radiotherapy.

## Introduction

Chondrosarcoma is a heterogenous group of malignant bone tumor arising from cartilage. It is the second most common primary bone sarcoma and accounts for 20–30% of bone tumors^1,2^. CHS is considered chemo- and radioresistant due to poor blood supply, slow dividing cells, dense extracellular matrix, hypoxic microenvironment, overexpression of proteins involved in drug resistance^3,4^ Consequently, radical surgery remains the primary curative treatment for chondrosarcoma, in case it is anatomically possible, but it often has major disabling consequences^3^. For this reason, radiation therapy is considered in case of incomplete resection, inoperable tumors or metastasis but high doses are needed which in the case of conventional photon therapy are difficult to be attained due to the limited tolerance of surrounding neural structures^2,5^. Ion beam therapy with carbon ions has shown improved results compared to conventional radiotherapy by enhancing local control^6^. Carbon ion therapy advantages include a finite range of dose deposition in surrounding tissue, given by the Bragg Peak, phenomena leading to a lower risk associated toxicity for a given dose, and a higher relative biological effectiveness benefit^7^. In vitro studies already proved a higher efficiency of carbon ions on different CHS cell lines^8–11^. However, even particle therapy induced low but significant damage in healthy tissue located at the entrance of the ion track before reaching the tumor. Therefore, the challenge to enhance the therapeutic effectiveness of radiotherapy remains despite the fact that local control is generally high with ion beam therapy. An emerging strategy is the use of nanoparticles (NPs) targeting the tumor in combination with particle therapy as it was proposed for photon therapy a decade ago^12^. Nanoparticle-based drug delivery system combined with radiotherapy could be a promising therapeutic approach. Here, iron oxide nanoparticles are used as doxorubicin carriers owing to their biocompatibility for normal healthy tissue that was demonstrated in clinics^13–16^ and for their magnetic transport capacity^17,18^. In addition, doxorubicin—based chemotherapy proved to be efficient only in some rare cases of CHS due to its systemic toxicity^19^. Therefore, the use of nanocarriers becomes an important alternative mechanism, as it can release drugs directly into tumor cells, sparing the healthy cells. Previously, we demonstrated the dual chemotherapy-radiosensitization efficiency toward photons of the core–shell iron oxide nanoparticles encapsulated in polyethylene glycol (IONP) loaded with doxorubicin (IONP_DOX_) in human cervical adenocarcinoma^20–22^.

The aim of the present study is to evaluate, for the first time, the biological and radiosensitizing effects of the IONP_DOX_ compound on human chondrosarcoma cells, in combination with high-LET carbon ion and conventional X-rays irradiation. The study was focused on cell survival, DNA damage, morphology of cell organelles and spectral profiles of nuclei. IONP_DOX_ nanocompound exerted radiomodulatory ability towards carbon ion and X-ray irradiation of CHS cells. Additionally, our work highlighted, for the first time, the spectral fingerprints of cell nuclei (mediated on its entire area) in CHS cells in the presence/absence of NPs followed by carbon ions/X-ray irradiation. Thus, new possible spectroscopic markers for NPs/ionizing radiation treatment have been brought to light.

## Results and discussion

Chondrosarcoma has a very poor prognosis due to its the high resistance to chemotherapy and radiotherapy that limits the therapeutic options. Consequently, multimodal treatment is recommended for improving CHS management^19^, tumor sensitization being a key feature in developing new therapeutical strategies.

Therefore, the main aim of our study was to evaluate whether the IONP_DOX_ nanoparticles in combination with photons and particle irradiation have a chemo- and/or radiosensitization potential on SW1353 chondrosarcoma cells. The SW1353 cell line was selected because it is known to be very radioresistant^2,23^ and represents the most prevalent CHS subtype, namely the conventional CHS (85%)^2^.

**Figure 1:**
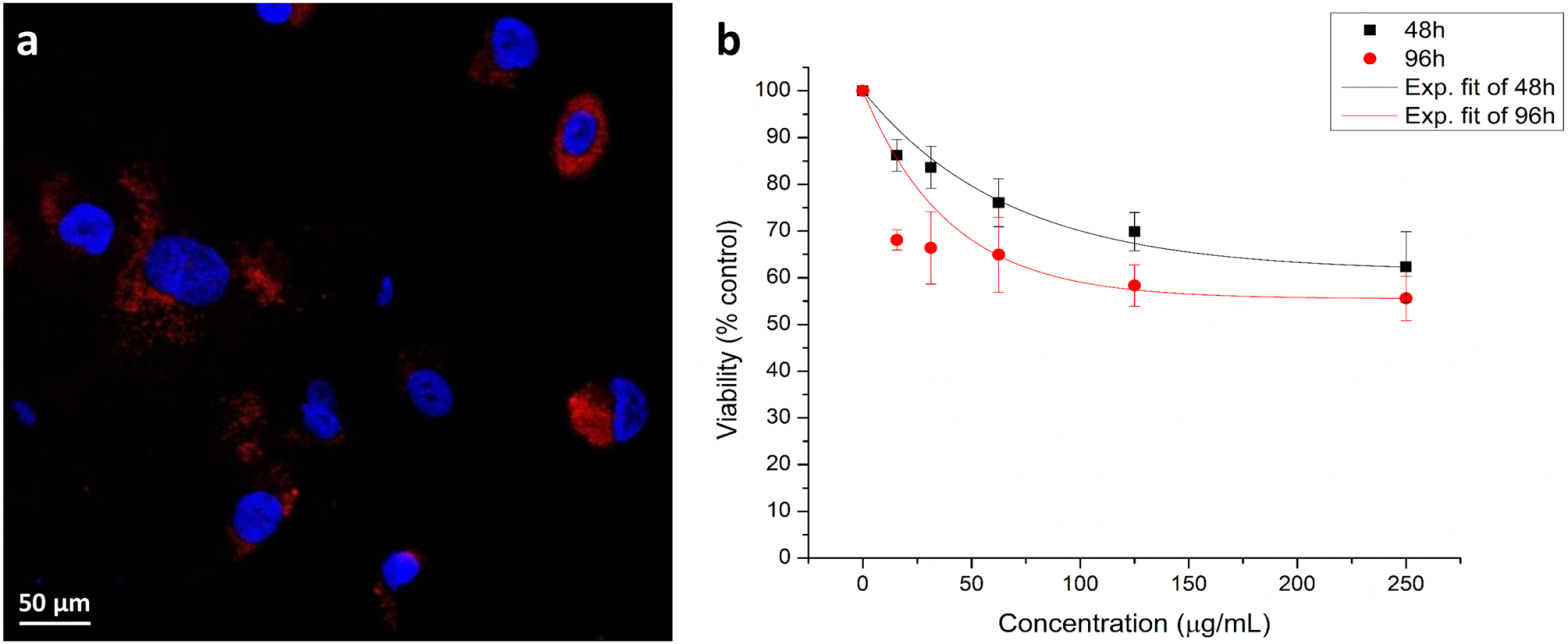
The biological effect of NPs on CHS cells. (**a**) IONP_DOX_ (administered concentration of 200 µg/mL) internalization in SW 1353 chondrosarcoma cells after 16 h of incubation; Fluorescence images: nuclei are stained with Hoechst (blue), red staining is fluorescence from incorporated DOX; (**b**) SW1353 viability after incubation during 48 and 96 h with IONP_DOX_ at different concentrations. Data are presented as mean ± SEM (n = 3).

First, we checked the uptake and retention of IONP in SW1353 chondrosarcoma cells using fluorescence microscopy imaging. The efficient internalization and retaining of nanoparticles in tumor cells are key factors for the radiosensitization effect^20,21^. The nanoparticles in chondrosarcoma cells were observed at the cytoplasmic level, in the peri-nuclear area of the cells. This internalization pattern is emphasized through the red-colored aggregates (Fig. 1a), and further observed also in hyperspectral images.

Quantitative determination of IONP_DOX_ cytotoxicity was done using MTT tetrazolium salt viability assay. The results showed a decrease of viability proportional to the IONP_DOX_ concentration (Fig. 1b). The decrease was statistically significant compared to control at both time intervals, 48 h and 96 h, for all the concentrations used, being higher for the 96 h incubation time. Considering the results obtained in this study and in the recent work published by our group^22^, we selected for following investigations, the nanoparticles concentration of 200 µg/mL.

**Figure 2:**
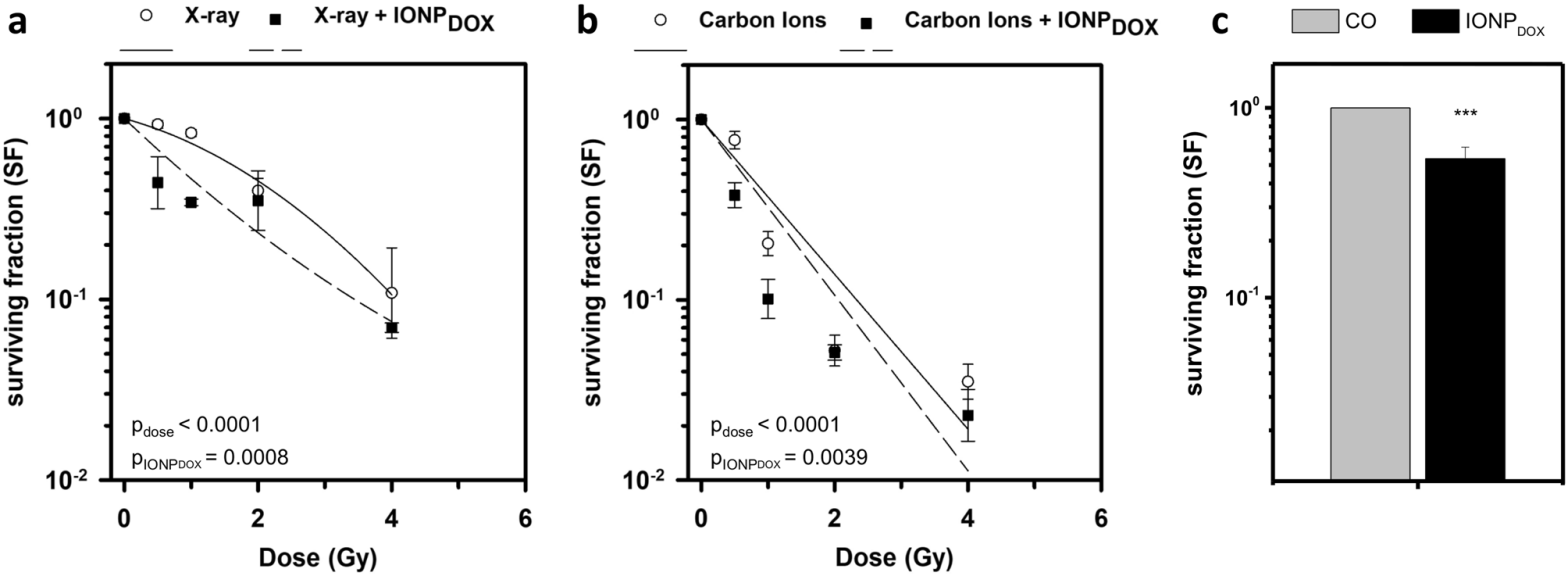
Clonogenic survival of SW1353 chondrosarcoma cells after exposure to 200 μg/mL IONP_DOX_ for 16 h, followed by 150 kV X-ray (**a**) or 95 MeV carbon ions treatment (**b**) and after exposure to 200 μg/mL IONP_DOX_ alone (**c**). Data are presented as mean ± SEM (n = 3). ***p < 0.001.

In order to study the radiomodulatory effect of IONP_DOX_, cell survival subsequent to IONP_DOX_ treatment followed by either X-ray or carbon ions irradiation was evaluated by the clonogenic assay; the survival fraction (SF) was plotted as a function of dose (Fig. 2a,b). Both types of irradiations in the presence of nanoparticles led to an amplified decrease of the radiation-induced clonogenic survival compared to the untreated samples (Fig. 2a,b). This enhanced reduction of the SF caused by NPs is dose dependent for both types of irradiations. The two-way ANOVA test proved a significant reduction of survival fraction by means of dose, as well as by nanoparticles (p_dose_ < 0.0001, p_NPs_ = 0.0008 in case of X-ray irradiation and p_dose_ < 0.0001, p_NPs_ = 0.0049 for carbon ions irradiation). The dose modifying factors corresponding to 0.1 survival fraction (DMF_SF=0.1_) expressing the overall IONP_DOX_ effect on the reduction of the survival have the following values: DMF_SF=0.1_ = 1.05 ± 0.03 for the treatment with NPs and X-rays irradiation, respectively DMF_SF = 0.1_ = 1.2 ± 0.1 for the NPs treatment followed by carbon ions irradiation. The addition of IONP_DOX_ before irradiation caused a significant decrease in cell survival at 0.5 Gy and 1 Gy for both X-ray and carbon ions irradiation (IONP_DOX_ + 0.5 Gy vs 0.5 Gy: p = 0.044 and IONP_DOX_ + 1 Gy vs 1 Gy: p < 0.0001—X-rays; IONP_DOX_ + 0.5 Gy vs 0.5 Gy: p = 0.0056 and IONP_DOX_ + 1 Gy vs 1 Gy: p = 0.041—carbon ions). IONP_DOX_ alone induced a significant cytotoxic effect on SW1353 cells, the survival decreasing to 0.52 ± 0.19 (Fig. 2c). IONP_DOX_—carbon ions combined treatment leads to a more pronounced sensitization effect in CHS than IONP_DOX_—X-rays, as expressed by DMFs. The radiosensitization effects in both cases are modest but significant and important; additionally, the controlled delivery of doxorubicin using biocompatible nanoparticles as carriers is expected to reduce the systemic toxic effects. Overall, the effect on cell death of both types of irradiation is enhanced when IONP_DOX_ compound is priorly added to the cells. It is known that iron oxide nanoparticles in presence of photon or particle radiation are capable to generate highly cyto- and genotoxic reactive oxygen species through the Fenton reaction and Haber–Weiss cycle^24^. In addition, the chemotherapeutic drug doxorubicin induces cytotoxicity through the generation of oxidative stress but also by DNA intercalation and inhibition of topoisomerase II^25^. This study demonstrates for the first time, the sensitizing potential of iron oxide nanoparticle-based drug delivery systems towards C-ions irradiation. To our knowledge, no report was made concerning the nanoparticle radiosensitization of CHS cells in case of carbon ions beams. Only few studies highlighted the efficacy of nanoparticles such as gold, gadolinium, tellurium, gadolinium-based gold to amplify the effects of medical carbon ions in other tumor cells types as reviewed recently^12,26^. The results presented here demonstrate the sensitizing potential of IONP_DOX_ in CHS cells following low energy X-ray irradiation and confirm our previous data obtained for other radioresistant tumor cell model (cervical adenocarcinoma HeLa)^21,22^. Recently, Alloy et al.^27^ proved the radiosensitization ability of gadolinium nanoparticles towards X-rays irradiation in two cell lines, a soft sarcoma (HEMC-SS cell line) and a conventional CHS (SW1353 cell line).

The potential genotoxic effects of the IONP_DOX_/irradiation/combined treatment on SW1353 chondrosarcoma cells was evaluated through micronuclei formation investigation. MN are recognized as being a valuable index of DNA damages generated by genotoxic agents, representing fragments or whole centric chromosome or chromatids resulting from unrepaired or mis-repaired double-strand breaks (DSBs)^28^.

**Figure 3:**
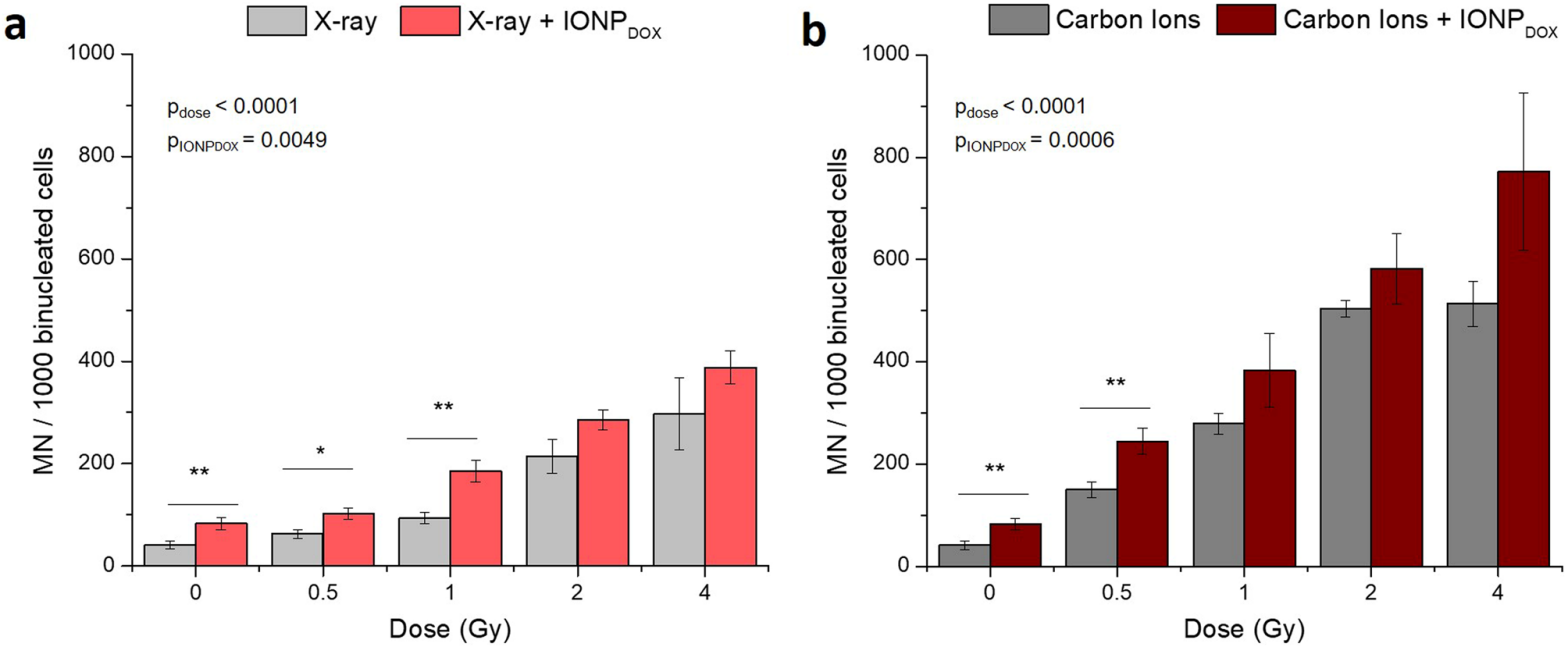
DNA damage measured as micronuclei in SW1353 chondrosarcoma cells after exposure to 200 μg/mL IONP_DOX_ for 16 h, followed by 150 kV X-ray (**a**) or 95 MeV carbon ions treatment (**b**). Data are presented as mean ± SEM (n = 3). p-values are presented as: *p < 0.05; **p < 0.01.

As can be noticed in (Fig. 3a,b), incubation with DOX-loaded IONP prior to both types of irradiations induced a statistically significant increase of micronuclei in chondrosarcoma cells in a dose dependent manner, as compared to the untreated samples (p < 0.001). The micronuclei yield increase is due not only to irradiation but also to the treatment with nanoparticles as showed by two-way ANOVA test (p_dose_ < 0.0001, p_NPs_ = 0.0006 in case of X-ray irradiation and p_dose_ < 0.0001, p_NPs_ = 0.0039 for carbon ions irradiation). Specifically, the dual treatment generated a significant increase in MN at 0.5 Gy and 1 Gy for X-ray (IONP_DOX_ + 0.5 Gy vs 0.5 Gy: p = 0.016 and IONP_DOX_ + 1 Gy vs 1 Gy: p = 0.002) and at 0.5 Gy for carbon ions irradiation (IONP_DOX_ + 0.5 Gy vs 0.5 Gy: p = 0.005). IONP_DOX_ alone determined a statistically significant enhancement of the micronuclei frequency (p = 0.0089) (Fig. 2c).

As expected, carbon ions induced a higher number of MN than X-rays. It is well known that carbon ions produce complex DNA double-strand break which are difficult to be repaired due to the high linear energy transfer^29^ and that MN are considered an indicator of radiation quality^28^.

All these results suggest that the accumulation of DNA damage contributes to the cytotoxicity mechanisms of the combined treatment, IONP_DOX_ followed by photon and/or particle therapy.

**Figure 4:**
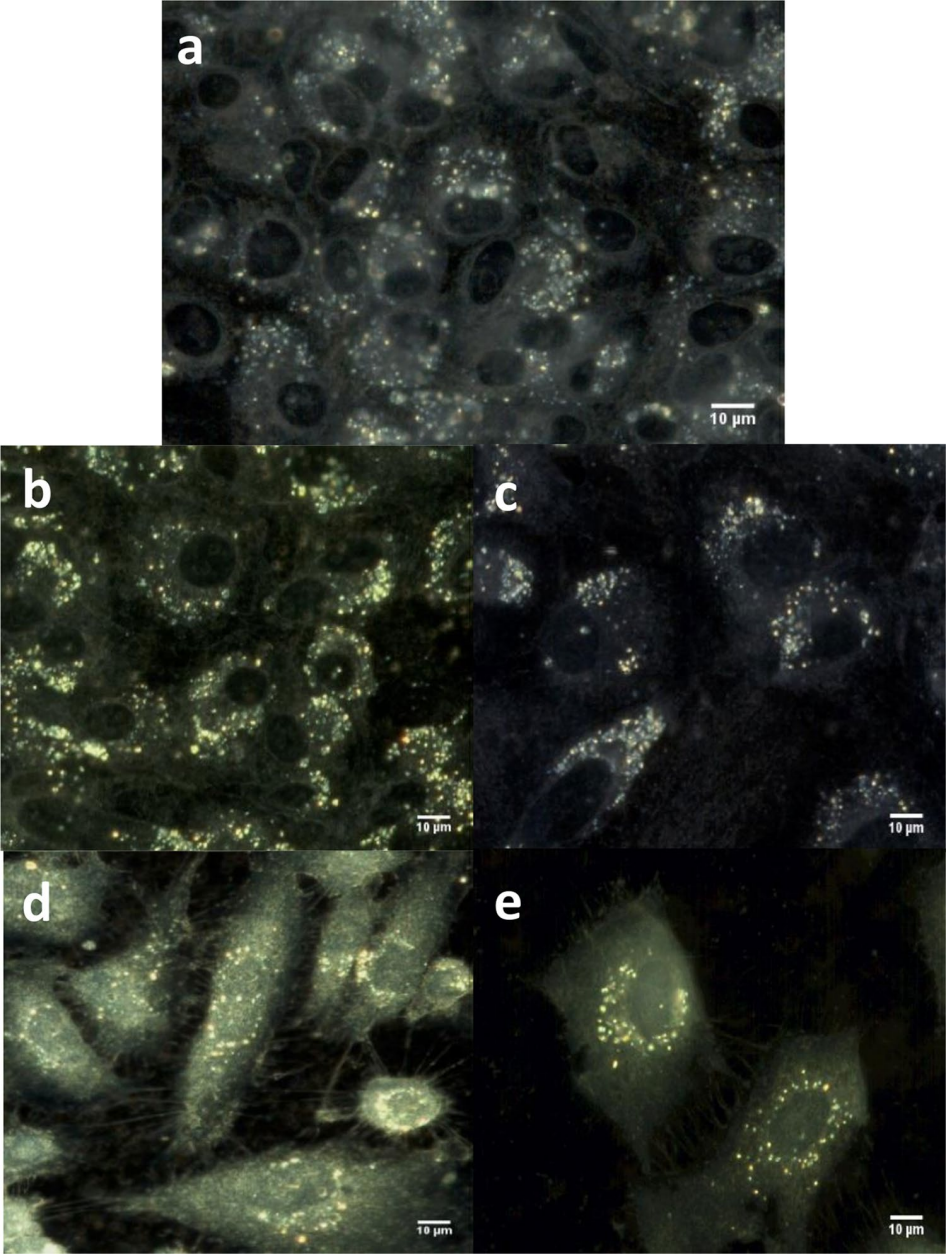
Hyperspectral images of CHS cells: incubated with NPs (**a**) treated with NPs and irradiated with carbon ions at 2 Gy (**b**) and 4 Gy (**c**) or with X-rays at 2 Gy (**d**) and 4 Gy (**e**).

**Figure 5:**
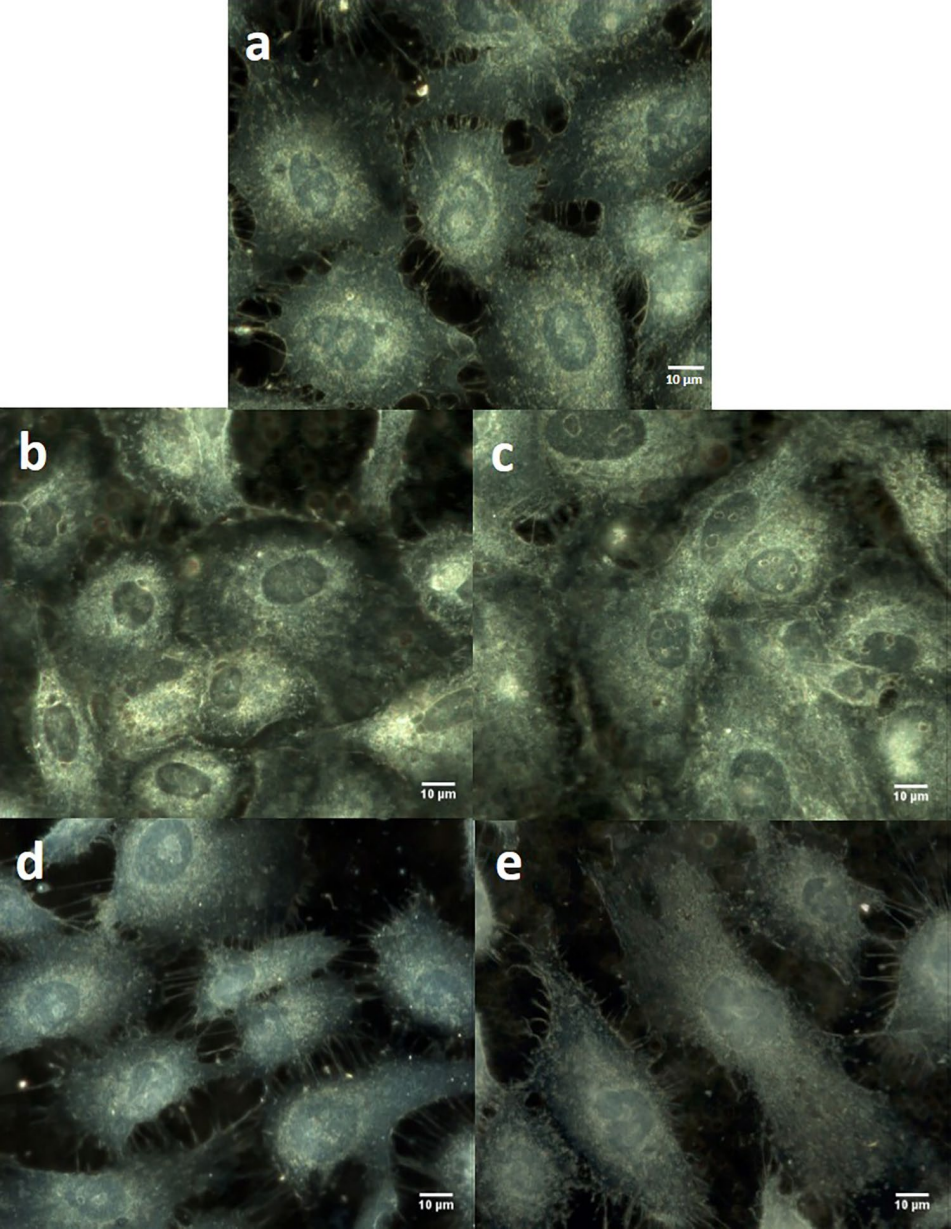
Hyperspectral images of CHS cells: untreated (**a**) irradiated with carbon ions at 2 Gy (**b**) and 4 Gy (**c**) irradiated with X-rays at 2 Gy (**d**) and 4 Gy (**e**).

To get an accurate understanding of chondrosarcoma cell behavior following the IONP_DOX_/irradiation/combined treatment, we continued our investigation by analysing the morphology of cell organelles, especially cytoplasm and nucleus, as well as the spectral profiles of nuclei. For this we used enhanced dark-field microscopy (EDFM) assembled with a hyperspectral imaging module. This imaging combination can be applied down to micro- and nanometer scales allowing analysis at single cell level with details from inside the nucleus^30,31^ or about nanoparticles internalization in cytoplasm^32,33^. Hyperspectral images were recorded on CHS cells treated with IONP_DOX_ alone (Fig. 4a) and in combination with carbon ions irradiation at 2 Gy (Fig. 4b) and 4 Gy (Fig. 4c) and X-rays irradiation respectively (Fig. 4d,e). The scattering intensities from NPs are very high and to avoid the CCD saturation of these pixels, the exposure time was chosen adequately; from this reason, the cytoplasm and nucleus have less intensity (compared with images of cells incubated without nanoparticles). We also recorded HSI on CHS cells untreated (Fig. 5a) and irradiated with 2 Gy (Fig. 5b) and 4 Gy (Fig. 5c) carbon ions and X rays respectively (Fig. 5d,e). This approach on the nuclei entire area represents a global analysis compared to our previous study at the pixel level^34^.

In all these HSIs without NPs, the nucleus structure and edges can be clearly seen by the advantage provided in EDFM to optically exclude the unscattered incident beam with the aim to enhance signal to noise ratio. The nuclei area is identified as regions of darker color for all cells. This aspect is already established in the literature^35^, concluding that dark field imaging under white light illumination can be used to identify the nucleus of the cells, without the need to use any chromatographic markers.

Morphologically, chondrosarcoma cells undergoing irradiation treatment (irrespective of nanoparticle addition) suffer an alteration in terms of density and thus spreading ability due to extended space on the 2D cell plate (Figs. 4b–e, 5b–e). The reduction of cell number in microscopy following irradiation treatment alone and respectively in the presence of nanoparticles was correlated with clonogenic survival results, showing both the cytotoxic effect of carbon ions at the selected doses, as well as the dual treatment with prior nanoparticle exposure. Additional to these observations, nanoparticles internalization can be easily highlighted in the correspondingly treated samples (Fig. 1), as high intensity round-shaped aggregates located in the peri-nuclear area, correlated with fluorescence microscopy images, as well as with previous results obtained on highly resistant cancer cells^20,21^.

**Figure 6:**
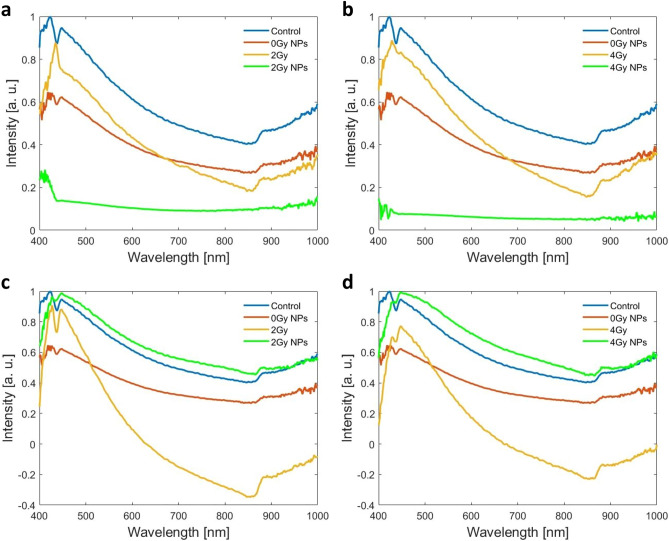
Spectral profiles as averaged values over the whole nucleus area on all nuclei from images of CHS cells: untreated and irradiated with carbon ions (**a,b**) and X-rays (**c,d**) in the absence or presence of NPs.

Enhanced dark-field optics coupled with hyperspectral sensors and modules significantly increases its applicability for cell biology, permitting spectral identification of specific signatures linked with quantitative analysis of cells organelles. The HSIs from Figs. 4 and 5 consist of hundreds of thousands of pixels; each pixel is stored as a three-dimensional (3D) hypercube data set containing information on two spatial dimensions and one spectral dimension. We exploited this advantage for accurate segmentation of cells nuclei using ROI tool on natural samples without any coloring marker. In the Fig. 6 are depicted the spectral signatures of cells nuclei in the analyzed situations (CHS cells: untreated, treated with NP_s_, irradiated with carbon ions at 2 Gy and 4 Gy and respectively with X-rays at the same doses, in absence or presence of NPs) as a continuous and complete record of the scattered radiation over the entire interval of wavelengths. All spectra were obtained on images following lamp correction subtraction and were normalized to maximum value.

Concerning the HSI spectral profile analysis of the nucleus area, both nanoparticles and/or irradiation treatment determined alterations of the spectral profile compared to untreated control cells (Fig. 6). The scattering spectrum of untreated control cells was mainly characterized by peak 1 located at 424 nm, peak 2 located at 445 nm, respectively a valley which was located at 436 nm; the ratio of intensities peak 1/peak 2 was greater than 1 (Fig. 6—blue spectrums). Following the incubation with 200 µg/mL nanoparticles, the intensity of the peak 1 drastically decreases compared to untreated control cells and it is replaced by a ruffling effect between 420 and 430 nm (Fig. 6—red spectrums). Also, a slightly small alteration of peak 2 intensity was also observed following incubation with nanoparticles, thus leading to a reduction in intensity of the valley at 436 nm (Fig. 6—red spectrums).

Irradiation alone induced significant alterations of the nuclei scattering spectrums, but these observations were highly dependent on the type of radiation. It seems that irradiation with carbon ions severely alters the location, shape and intensities of SW1353 cells’ characteristic spectral peaks (Fig. 6a,b—yellow spectrums). Thus, following irradiation with carbon ions alone, peak 1 at 424 nm shifts towards 430 nm wavelength, while peak 2 at 445 nm completely disappears. Thus, the valley at 436 nm also disappears (Fig. 6a,b—yellow spectrums). Depending on the dose of radiation, scattering spectrums of nuclei of cells exposed to 2 Gy carbon ions alone showed a sharp peak 1 at 430 nm with high intensity, while scattering spectrums of nuclei of cells exposed to 4 Gy carbon ions alone showed a wider peak with lower intensity compared to 2 Gy (Fig. 6a,b—yellow spectrums).

Irradiation with 2 Gy X-rays alone increases both the intensity of peak 1, as well as peak 2. However, irradiation with 4 Gy X-rays alone severely decreases the intensity of peak 1, while the intensity of peak 2 remains almost the same (compared to untreated control cells) (Fig. 6b,c—yellow spectrums).

The addition of nanoparticles followed by carbon ions irradiation severely altered the scattering spectrums of the nuclei in treated cells compared to controls: a ruffled peak at about 420 nm was still observed for 2 Gy dose, while this completely disappears at 4 Gy dose.

The nucleus is the most prominent organelle housing the cell’s DNA, and an indicator of the effects of ionizing radiation. X-rays and carbon ions generate severe alterations of the DNA composition by ionization of the atoms of the cell’s genetic material. We analyzed for the first time with the hyperspectral module under dark field microscopy, irradiated cells and obtained spectral profiles averaged over the entire area of the nucleus (thousands of pixels). These are different in all the cases studied: non-irradiated, irradiated with X-rays or with carbon ions, in combination with nanoparticles or not. Being averaged over the entire area, they include information about the intensities at each wavelength (400–1000 nm) and from each pixel, therefore also about all alteration centers (micronuclei) that are spread over the entire area of a nucleus. The disappearance of the second maximum (445 nm orange curve Fig. 6a,b) in the case of irradiation with carbon ions and the inversion of the intensities (orange curve Fig. 6d) indicate that the two types of radiation modify the nuclei differently. The effect of the alteration is even more obvious in the case of cell nuclei subjected to both effects (NPs and irradiation), by the fact that the spectral profiles no longer retain any of the maxima from the non-irradiated nuclei in the case of carbon ion irradiation (green curves in Fig. 6a,b) and the almost disappearance of the first maximum in the case of X-ray irradiation (green curves in Fig. 6c,d). Comparing spectral profiles from control nuclei with the nuclei of cells incubated with NPs but non-irradiated, we can say that in this case too there are changes, but not as obvious as in the case of cell nuclei subjected to both effects.

This change in scattering effect from the pixels inside nuclei can be correlated with biological events underwent by cells, which influence its chemical composition. We did not consider the cytoplasms in this study because in cells exposed to nanoparticles, their accumulation in the cytoplasm influences its spectral behavior; scattering effect can be shadowed by the presence of the iron oxide nanoparticles, which show an absorption effect of light with a large peak centered at 370 nm^36^.

Nevertheless, these alterations in the nuclei scattering spectrums of the treated cells are correlated with an increase in the micronuclei number. The fragmentation or loss of chromosomes affects the DNA content in the nuclear compartment, as well as the (bio)chemical balance. It has been previously shown that changes in the molecular composition of DNA determine alterations of the molecules spectrums^37^.

Scattering radiation from subcellular organelles are mainly dependent on their molecular composition^31,38^. For example, its examination has revealed great potential in the diagnosis of cancer, recognizing protein biomarkers and genomic alterations on individual tumor cells in vitro^38,39^.

Until now, the spectral fingerprint of cells nuclei has been studied in the HIS literature with the aim to distinguish normal and cancerous cells but using coloring markers: hematoxylin–eosin-stained preparations^40,41^, or fluorescent markers^42^. In these studies, 20× or 40× microscope objectives were used leading to an overall imaging of the nuclei, without detailing the components inside the nucleus.

The novelty of our work is that it presents for the first time the spectral fingerprint of cells’ nuclei in SW1353 line as averaged values on all nuclei area (thousands of pixels) on unstained samples from high spatial resolution images formed from scattered radiation. In this HSI study, the spectral profiles from control nuclei and the spectral profile changes when the cells are exposed to ionizing radiations or/and incubated with IONP_DOX_ were highlighted to identify possible spectroscopic markers of nanoparticles and ionizing radiation treatment. Thus, we demonstrated that cells nuclei exposed to different external conditions exhibited distinctive spectral features as well as cells nuclei alteration due to ionizing radiations or nanoparticles that can be easily observed in HSI from unstained samples. Hyperspectral analysis is a promising way to analyze fast the information given in the tumor cells and by this could be important in tumor tissue management.

## Materials and methods

### Cell culture

The chondrosarcoma cell line SW1353 (CLS Cell Lines Service GmbH, Eppelheim, Germany) was cultured in Dulbecco’s modified Eagle’s High-Glucose Medium (PAN Biotech, Aidenbach, Germany) supplemented with 10% Fetal Bovine Serum (FBS, EuroClone,Via Figino, Italy), 5% L-Glutamine (Sartorius, Beit Haemek, Israel) and 1% Penicillin/Streptomycin solution (Capricorn Scientific GmbH, Ebsdorfergrund, Germany). Cells were maintained at 37 °C in a humidified incubator with 5% CO2.

### Treatment of chondrosarcoma cells with nanoparticles

**Figure 7:**
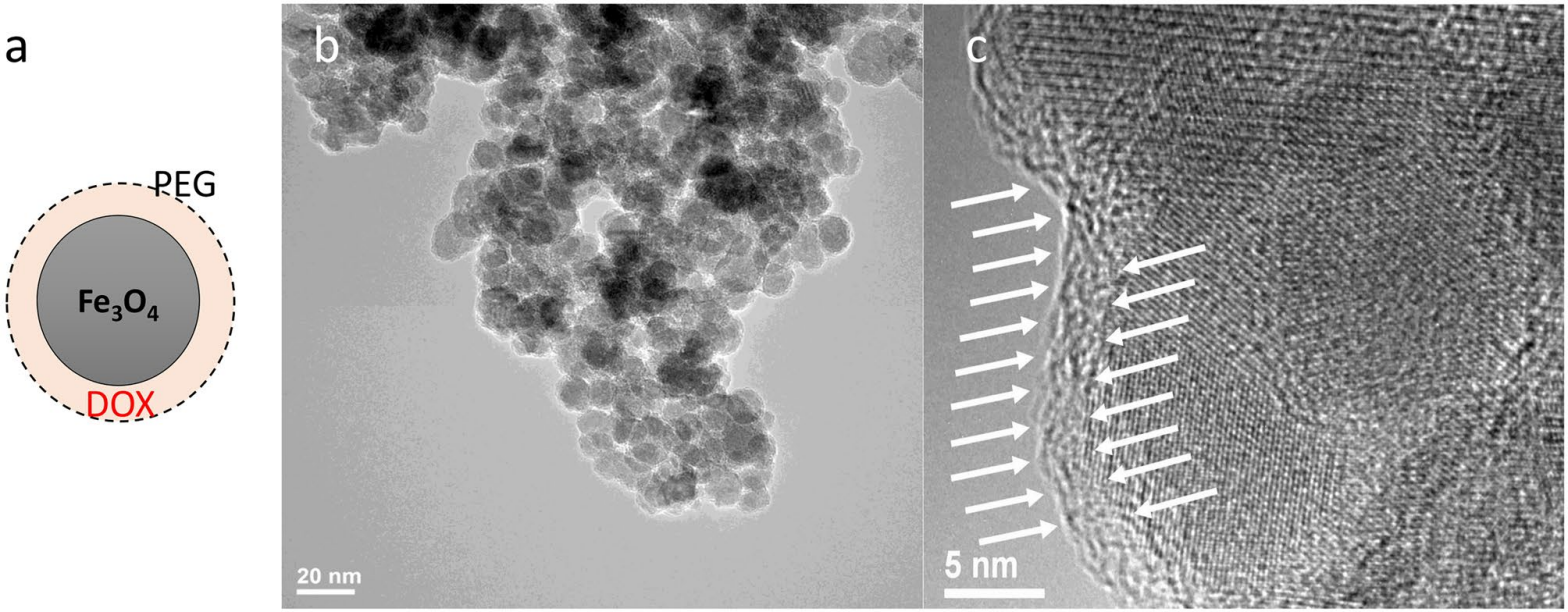
Morphology of doxorubicin-iron oxide nanoparticles: (**a**) core–shell schematic representation of doxorubicin-loaded iron oxide nanoparticles conjugated with polyethylene glycol with 6000 Da molecular weight, (**b**) transmission electron microscopy image illustrating the round-shape morphology of the iron oxide nanoparticles, (**c**) detailed high resolution transmission electron microscopy image of the PEG shell (white arrows).

The nanoparticles used in the present work, namely core–shell polyethylene glycol (6 kDa)-encapsulated iron oxide nanoparticles (IONP) with a hydrodynamic diameter of 160 nm and loaded with doxorubicin (IONP_DOX_) were previously synthetized and characterized in our studies^20^. The doxorubicin-loaded iron oxide nanoparticles, characterized in terms of size, showed a mean hydrodynamic diameter of 369.1 and a Polydispersity Index of 0.238, proving to be a monodisperse suspension^20^. The Fig. 7 illustrates the morphology of the doxorubicin-iron oxide nanoparticles as described previously^20^. Such as, it could be observed a schematic representation of the core–shell morphology of the polyethylene glycol conjugated iron oxide nanoparticles loaded with doxorubicin (a). The real morphology of these nanoparticles is emphasized in Fig. 7a, showing a round shape of the nanoparticles with medium degree of aggregation, mostly given the evaporation of the water following the placement of the nanoparticles suspension on the carbon grid. Figure 7c gives a high-resolution TEM image of the core–shell nanoparticles and emphasized the polymer shell at the exterior of the nanoparticles (white arrows). Concerning the stability of the doxorubicin-loaded nanoparticles this has also been discussed in Popescu et al.^20^ where the release of the drug was investigated following incubation in three biologically relevant media (pH 7.4/6.5/4.8) showing an exponential delivery until about 20 h, which was not significantly influenced by pH. For in vitro studies, the chondrosarcoma cells were seeded at different concentrations depending on the investigation method and incubated for 4 h to allow attachment. This was followed by the replacement of the culture medium with fresh medium containing IONP_DOX_ at a concentration of 200 μg/mL and incubated for 16 h.

### Detection of IONP internalization

Uptake and retention of IONP in SW1353 was assessed using a previous protocol^20^. Chondrosarcoma cells were seeded in 24 well plates (TPP Techno Plastic Products AG, Trasadingen, Switzerland) at a concentration of 10^5^ cells/well and treated as described above. Afterwards, cells were washed three times with PBS, detached and centrifuged at 1700 rpm for 5 min, to eliminate the nanoparticles that were not interacting directly with the cells. The supernatant was replaced with nanoparticle free fresh medium and the cells were seeded on glass coverslips at a density of 5 × 10^4^ cells/slide and incubated for an additional 24 h. After this time, the coverslips were washed with PBS and fixed with 4% polyformaldehyde for 10 min. Afterwards, the samples were incubated for 10 min with Hoechst (Invitrogen, Thermo Fisher Scientific, Waltham, USA) at a concentration of 1 μg/mL. Following staining, coverslips were washed with PBS and mounted on a slide with glycerol. Imaging was done using the epifluorescence microscope Olympus BX-51 (Olympus, Germany) taking advantage of DOX autofluorescence, while Hoechst counterstaining of the nuclei was applied.

### Proliferation assay

Cellular viability of chondrosarcoma treated with nanoparticles was determined through the MTT assay. SW1353 cells were seeded in 96 well plates (TPP Techno Plastic Products AG, Trasadingen, Switzerland) at a concentration of 3000 cells/well for 48 h assessment and 1000 cells/well for 96 h and treated with nanoparticles of concentrations between 15 and 250 μg/mL, with 3 cell-free blanks for each concentration, as described above. Following the incubation period, cells were washed 3 times with PBS and incubated with 100 μL of complete medium with 10% MTT (Sigma-Aldrich Chemie GmbH, Burlington, MA, United States) for 3 h in standard cell culturing conditions. Following incubation, the medium was removed and the resulting formazan crystals were solubilized using DMSO (Sigma-Aldrich Chemie GmbH, Burlington, MA, United States). The supernatant absorbance was measured at 570 nm using a spectrophotometer (Mithras, Berthold technologies, Bad Wildbad, Germany).

### Cells irradiation

For irradiation with either X-ray or carbon ions, chondrosarcoma cells were seeded at 5 × 10^5^ concentration in 25 cm^2^ flask (USA Scientific, Inc, Ocala, FL, USA) and treated as described in “Treatment of chondrosarcoma cells with nanoparticles” section. After the 16 h incubation, cells were washed 3 times with PBS and fresh culture medium was added to the recipient.

The SW1353 cells were irradiated using the XSTRAHL XRC 160 machine of IFIN‐HH, generating 150 kV X-rays at a dose rate of 1 Gy/min, with a 25 cm^2^ irradiation field.

For carbon ions exposure, irradiation was achieved by using the high-energy beam line with carbon ions of 95 MeV/A and a LET of 73 keV/μm from the IRABAT facility at GANIL (Caen, France). This LET was obtained from a native beam of carbon ions by using a methyl polymethacrylate (PMMA) filter located between the beam exit area and the irradiation sample. This beam time was obtained under the experiments nr P1304-H of the iPAC 2021 call.

Cells with and without IONP were exposed to 0, 0.5, 1, 2 and 4 Gy, where 0 Gy represents the sham control.

### Colony formation assay

The colony formation assay was performed immediately after irradiation, using cells prepared in 25 cm^2^ flasks as described above. For each experimental condition, the cells were detached, counted 2 times and seeded at different densities, in regard to irradiation dose and IONP treatment (800 to 7000 cells/well), in 2 mL medium, in 6 well plates. The plates were incubated under standard conditions for 14 days, followed by staining with a solution of crystal violet (Sigma-Aldrich Chemie GmbH, Burlington, MA, United States) and methanol/ethanol, only colonies with at least 50 cells were counted. After counting the colonies, the plating efficiency (PE) and the survival fraction (SF) were calculated according to the following formulas:

$$PE=\frac{\text{number of counted colonies}}{number\, of \,seeded \,cells} \times 100 \hspace{2 mm} SF=\frac{\text{number of counted colonies}}{number \,of\, seeded \,cells} \times PE.$$

The survival fraction of cells has been reported to control cells (non-irradiated).

The surviving fraction (SF) was fitted with the linear-quadratic model (ln(SF) = − (αD + βD^2^)) for X-ray and the linear model (ln(SF) = (a*D)) for carbon ions using the non-linear regression tool of SigmaPlot 15 (Systat Software GmbH, Erkrath, Germany)^43^.

### Micronucleus assay

After irradiation, for each experimental condition, the cells were detached, counted and seeded at 10^4^ density on 10 mm coverslips. Cytochalasin B (Sigma-Aldrich Chemie GmbH, Burlington, MA, United States), at a concentration of 3 μg/mL in culture medium, was added 20 h prior to fixing the cells. After the incubation time, cells were fixed using an acetic acid-methanol solution (1:9) and stained using acridine orange (Sigma-Aldrich Chemie GmbH, Burlington, MA, United States) at a concentration of 10 µg/mL in PBS. For visualization, an epifluorescence microscope Olympus BX-51 (Olympus, Germany) was used. To determine the number of micronuclei we scored MN in 1000 binucleated cells using the criteria described by Fenech^44^.

### Hyperspectral image acquisition

The cell samples for hyperspectral image acquisition were previously treated with nanoparticles and irradiated as similar for the clonogenic assay as above. Following radiation, cells were detached and seeded onto 10 mm glass slides at a concentration of 10,000 cells/slide and allowed to attach for another 24 h. Following incubation in standard conditions, cells were fixed with 4% paraformaldehyde solution and mounted on a microscope slide using glycerol. All samples were prepared without any contrast agents or other chemical chromatographic markers.

Hyperspectral images (HSI) acquired in enhanced dark field microscopy were obtained using a CytoViva^R^ system (CytoViva Inc., USA). A white light source (FiberLite DC-950, 150W quartz halogen aluminum reflector, Dolan Jenner Industries, USA) was coupled with a liquid-core fiber (for reduced thermal noise) to illuminate the condenser (patented cardioid shape, oil immersed). In this way, very narrow oblique illumination of the sample was obtained (no direct light enters in the objective), to provide high-contrast images of nanometer details on dark background. This configuration improves signal-to-noise ratio by over ten times compared with a standard dark-field microscope. A 60× oil immersed microscope objective (1.4 NA) forms the image of sample details on the diffraction grating of a spectrophotometer working in transmission (ImSpectrum V10E, SPECIM, Spectral Imaging Ltd., Finland, range 400–1000 nm) placed in front of the color CCD (1392 × 1040 pixels resolution, 7.3 to 13.5 fps, 6.45 × 6.45 µm pixel size, 5 µs–60 s exposure time range). It thus allows a spectral resolution of 1.28 nm in 400–1000 nm range of hyperspectral images. The HSI are obtained by automatic transversal scanning of each line (NanoScanZ Prior Scientific Instruments Ltd, UK).

HSI were acquired using slides with cells incubated with/without NPs and irradiated with 0 Gy, 2 Gy and respectively 4 Gy from carbon ions or X-Ray sources.

The dedicated software ENVI was used to display the acquired images; special analysis tools provide spectral profiles at single pixel level or on regions of interest as graphical representation of the scattered intensity distribution at each wavelength. Cell nuclei segmentation was performed using ROI tool. Specific scattering spectra were collected from these selected pixels and is plotted as average values on all intensity values of each pixel and on all nuclei from an image.

### Statistical analysis

The data obtained from at least 3 experiments, each experiment having 3 replicas for each condition, are expressed as the mean ± SEM. Statistical analysis was performed using two-way ANOVA (GraphPad Prism 8.2, La Jolla, CA, USA) or Student t-test. Results are considered statistically significant when p < 0.05 (*p < 0.05, **p < 0.01, ***p < 0.001, ****p < 0.0001).

## Data Availability

The data sets used and analysed in this present work are available from the corresponding authors upon reasonable request.
